# Dynamic FDG-PET Imaging to Differentiate Malignancies from Inflammation in Subcutaneous and In Situ Mouse Model for Non-Small Cell Lung Carcinoma (NSCLC)

**DOI:** 10.1371/journal.pone.0139089

**Published:** 2015-09-30

**Authors:** Zhen Yang, Yunlong Zan, Xiujuan Zheng, Wangxi Hai, Kewei Chen, Qiu Huang, Yuhong Xu, Jinliang Peng

**Affiliations:** 1 School of Biomedical Engineering, Shanghai Jiao Tong University, Shanghai, China; 2 Department Automation, School of Electrical and Information, Sichuan University, Chengdu, Sichuan, China; 3 School of Pharmacy, Shanghai Jiao Tong University, Shanghai, China; 4 Banner Good Samaritan Medical Center, Phoenix, Arizona, United States of America; Fondazione IRCCS Istituto Nazionale dei Tumori, ITALY

## Abstract

**Background:**

[^18^F]fluoro-2-deoxy-D-glucose positron emission tomography (FDG-PET) has been widely used in oncologic procedures such as tumor diagnosis and staging. However, false-positive rates have been high, unacceptable and mainly caused by inflammatory lesions. Misinterpretations take place especially when non-subcutaneous inflammations appear at the tumor site, for instance in the lung. The aim of the current study is to evaluate the use of dynamic PET imaging procedure to differentiate in situ and subcutaneous non-small cell lung carcinoma (NSCLC) from inflammation, and estimate the kinetics of inflammations in various locations.

**Methods:**

Dynamic FDG-PET was performed on 33 female mice inoculated with tumor and/or inflammation subcutaneously or inside the lung. Standardized Uptake Values (SUVs) from static imaging (SUVmax) as well as values of influx rate constant (*Ki*) of compartmental modeling from dynamic imaging were obtained. Static and kinetic data from different lesions (tumor and inflammations) or different locations (subcutaneous, in situ and spontaneous group) were compared.

**Results:**

Values of SUVmax showed significant difference in subcutaneous tumor and inflammation (*p*<0.01), and in inflammations from different locations (*p*<0.005). However, SUVmax showed no statistical difference between in situ tumor and inflammation (*p* = 1.0) and among tumors from different locations (subcutaneous and in situ, *p* = 0.91). Values of *Ki* calculated from compartmental modeling showed significant difference between tumor and inflammation both subcutaneously (*p*<0.005) and orthotopically (*p*<0.01). *Ki* showed also location specific values for inflammations (subcutaneous, in situ and spontaneous, *p*<0.015). However, *Ki* of tumors from different locations (subcutaneous and in situ) showed no significant difference (*p* = 0.46).

**Conclusion:**

In contrast to static PET based SUVmax, both subcutaneous and in situ inflammations and malignancies can be differentiated via dynamic FDG-PET based *Ki*. Moreover, Values of influx rate constant *Ki* from compartmental modeling can offer an assessment for inflammations at different locations of the body, which also implies further validation is necessary before the replacement of in situ inflammation with its subcutaneous counterpart in animal experiments.

## Introduction

[^18^F]fluoro-2-deoxy-D-glucose positron emission tomography (FDG-PET) is one of the most widely used imaging techniques for detecting and staging tumors, as elevated glucose metabolism is indicative of malignancies [1]. However, regardless of its high accuracy and sensitivity, high FDG uptake is not tumor-specific. High level of FDG uptake can also be detected in normal tissues or benign lesions such as inflammation [2], causing false-positive results and misinterpretation for clinical diagnosis. Moreover, such false-positive issue is one of the major problems in the clinical staging of non-small cell lung carcinoma (NSCLC) [3]. Using Lewis Lung Carcinoma (LLC) bearing mice and different kinds of inflammatory models, our current study aimed to determine whether certain parameters estimated from the kinetic modeling approach can serve as a useful and more specific index in differentiating inflammations from malignancies in NSCLC, and in differentiating variation of inflammatory processes.

Tremendous efforts have been reported in the literature to deal with such FDG-PET false-positive issue, with different tracers, such as radiolabeled amino acid O-(2-18F-fluoroethyl)-L-tyrosine (FET) [4], 3'-deoxy-3'-(18) F-fluorothymidine (FLT), ^11^C-choline and ^11^C-methionine [5]. Compared to FDG, FET’s uptake by tumor cells is more stereospecific. However, FET’s tumor specific characteristics may differ from species. Further clinical studies need to determine whether it can be used in patients more commonly in clinical settings [6]. FLT depends on tumor cell proliferation and it is more tumor-specific than FDG. However, FLT has lower sensitivity, making it difficult to visualize the lesion [7–9]. It also has high physiological uptake due to increased perfusion and vascular permeability [10]. Therefore, the use of FLT in place of FDG for staging tumors is not yet feasible. In addition to these ^18^F labeled tracers, ^11^C-choline and ^11^C-methionine have great limitations due to the short half-life of ^11^C.

In parallel to introducing new tracers, researchers have also proposed different analysis methods to analyze FDG-PET data to increase the sensitivity in differentiating inflammations from malignancies. In most static FDG-PET studies, Standardized Uptake Value (SUV) is used as a semi-quantitative index in conjunction with the visual interpretation [11]. As pointed out earlier, SUV doesn’t help in discriminating inflammation from tumors, because the intensity of FDG uptake can resemble that of malignancy. SUV is also influenced by many factors such as the length of uptake period, body composition, partial volume effects, etc. [12]. And it’s especially not adequate when quantifying uptake in lungs because of the ineffectiveness of SUV correction [13].

The issue of how to avoid false-positive diagnosis in the lungs has been a serious problem encountered by radiologists for years. The most popular way to diagnose thoracic diseases is still by static PET imaging, which is difficult to identify changes of FDG uptake in the lung visually, causing misdiagnosis when it happens to infectious diseases or tumors with low glycolytic activity. Proper interpretation only happens when an experienced physician is aware of certain conditions [14].

Another semi-quantitative SUV based method was the dual time point imaging procedure. It was shown that FDG accumulations in some tumors rise with time while uptake in benign lesions decreases [15, 16]. However accumulations in malignancies over time were not observed for all tumors and the inflammatory process seems to be more complicated than a monotonic decreasing function [17].

Absolute PET quantification of physiological parameters via tracer kinetic modeling has been reported in numerous studies. It uses dynamic PET acquisition consisting of a series of frames over continuous time intervals. Data from different frames are reconstructed to form a set of images independently, which can be used to estimate physiological parameters [18]. Compared to static and dual time PET imaging, dynamic PET is expected not only to be more helpful in understanding the pathophysiological mechanisms of diseases, but also in extracting physiological or biochemical parameters via tracer kinetics. These parameters are often crucial for interpreting dynamic PET data and to better discriminate inflammation from malignancies [19].

In spite of the fact that compartmental modeling is the most commonly used method to provide quantitative information for PET studies, the drawback of this method is the requirement of the plasma Time Activity Curve (TAC) as the input function for the compartmental model, which is conventionally measured by arterial blood sampling [20]. Although blood sampling is considered a gold stand because of its high accuracy, it provides challenges and additional risks. Subsequent studies have solved the problem by introducing noninvasive methods by investigating the use of image-derived input functions [21, 22].

In this study, we performed dynamic PET imaging with the image-derived input function in different mouse models and analyzed both static and dynamic results by obtaining semi-quantitative parameters (SUVs) and quantitative parameters to determine whether dynamic PET imaging is a better way to differentiate inflammatory lesions from malignancies. Moreover, we compared the kinetics of different locations of the lesions to investigate whether a potential new assessment for tumor or inflammation can be presented to minimize the misinterpretation during diagnostics.

## Materials and Methods

### Animal Preparation and Experimental Groups

The protocol for our study was approved by Animal Studies Committee at Shanghai Jiao Tong University. Experiments were performed on 33 female C57/BL mice (8–10 weeks) weighing 21.1 ± 3.9 g, and were housed in air-filtered, temperature-controlled units with access to food and water ad libitum. Animals were randomly divided into 6 groups: (a) subcutaneous tumor inoculation group (n = 5), (b) in situ tumor inoculation group (n = 5), (c) subcutaneous inflammation group with tumor (n = 4) (d) subcutaneous inflammation group without tumor (n = 4), (e) in situ inflammation group (n = 9) and (f) spontaneous liver inflammation group (n = 6). The specific locations of tumor and inflammation in different groups are shown in Table 1.

**Table 1:** Location of Tumor and Inflammation in Different Experimental Groups.

Group	Location of tumor	Location of inflammation
Subcutaneous tumor	Axillary area	N/A
In situ tumor	Lung	N/A
Subcutaneous inflammation **with** tumor	Axillary area	Gastrocnemius muscle (right hind leg)
Subcutaneous inflammation **without** tumor	N/A	Gastrocnemius muscle (right hind leg)
In situ inflammation	N/A	Lung
Spontaneous liver inflammation	N/A	Liver

### Tumor Model

The experiment was implemented on Lewis Lung Carcinoma (LLC) bearing mice. LLC is a kind of tumor cell (NSCLC) originated spontaneously as a carcinoma of the lung of a C57/BL mouse. Cells were cultured in DMEM supplemented with 10% fetal calf serum at 37°C in a humidified atmosphere containing 5% CO_2_.

To obtain subcutaneous tumors, 2×10^6^ cultured tumor cells in 50 μL cell suspension were inoculated under the skin. For in situ tumors, 1×10^7^ tumor cells in 30 μL cell suspension mixed with 20 μL Matrigel (Promega, USA) were inoculated inside the lung. 2–3 weeks after tumor cell inoculation (subcutaneous tumor volume 319.5 ± 115.1 mm^3^), animals were sent for PET scan.

### Inflammatory Model

The subcutaneous inflammation was induced by inoculation of turpentine oil on the gastrocnemius muscle of the right leg (0.1 mL). In situ inflammation of lung was implemented by dripping of 50 μL (1 g/L) Lipopolysaccharide (LPS, Sigma Chemical Co, USA) into the trachea. PET scan was carried out 4 days after inoculation. For spontaneous inflammation group, experimental mice were put along with mice infected by Mice Hepatitis Virus (MHV) to induce spontaneous liver inflammation.

### PET Data Acquisition and Reconstruction

All animals were left fasting overnight the day before acquisition. All animals were anesthetized with inhalant anesthesia (1%-2% isoflurane in 100% oxygen) using a nose cone. Before acquisition, a 29-gauge needle connected to a catheter was placed into the lateral tail vein for FDG tracer administration. After anesthesia, the animal was placed in a prone position on the platform of the scanner and put in the center of the field view by laser beam calibration. All data acquisitions were initiated before the tracer injections. After the scan was started, a bolus of FDG (5.55 ± 0.814 MBq) was injected through the tail-vein catheter manually, the error caused by the injector and the catheter was calculated by subtracting the remaining dose. A 60 min dynamic imaging was acquired on a PET scanner (Siemens Inveon) followed by a 20 min CT scan at 2 bed positions.

The acquired list-mode data were reconstructed with 3-dimensional ordered-subset expectation maximization (OSEM) algorithm [23] using the software of Siemens Inveon Acquisition Workplacce with a framing protocol of 2×1.5 s, 10×0.5 s, 8×5 s, 1×20 s, 1×30s, 1×75s, 1×120s, 1×150 s, 1×400 s, 1×600 s, 1×750s, and 1×900 s.

### PET Data Analysis

All the reconstructed data was analyzed using the following steps: Definition of Region of Interest (ROIs), visual and semi-quantitative analysis of static images via Standardized Uptake Values (SUVs), Determination of plasma input functions and output functions of different lesions, obtaining the influx rate constant (*K*_*i*_) through compartmental modeling.

### Region of Interest Definition

Three-dimensional ellipsoid ROIs were drawn manually over the left ventricle (blood-pool), tumor, inflammatory tissue and liver with a landmark using the software of Siemens Inveon Research Workplace. Localization of FDG accumulation of focal tissue was performed visually with the help of the combination of PET and CT images. For static analysis, the maximum value of SUV within each ROI was selected as SUVmax. For dynamic analysis, a new ROI was generated automatically to replace the original one using a region growing method [24] which only included pixels within a range (± 40% of the average pixel value) to reduce the error.

### Plasma Input Function

Since the input function can be retrieved from the image data with good accuracy instead of blood sampling [25], Time Activity Curve (TAC) of the blood-pool from the left ventricle was extracted as the image-derived input function.

The input function was calibrated by a time-dependent plasma-to-whole-blood concentration ratio R_PB_ (t). R_PB_ (t) was defined as

$$R_{PB}(t)\;=\;0.432e^{-0.168t}\;+\;1.158$$ (1)

where time t was given in minutes. R_PB_ (t) was previously calculated by Wong et al. through estimating concentration ratios between plasma and whole-blood samples collected during PET acquisition at different times *t* (min) through nonlinear least-squares fitting [26].

### Static Analysis

The uptake of FDG on the last frame (at 45–60 min) of dynamic scans was used for static data analysis. Visual assessment was first performed on static images for determining the right anatomical location of focal tissues as well as defining ROIs. Tracer accumulation in the ROIs was reported as the Standardized Uptake Value (SUV)

$$SUV=\frac{Radioactivity\;Concentration\;in\;Region\;of\;Interest\;(MBq/mL)}{Injected\;Dose\;(MBq)/Weight\;of\;Animal\;(g)}$$ (2)

### Kinetic Analysis

**Fig 1:**
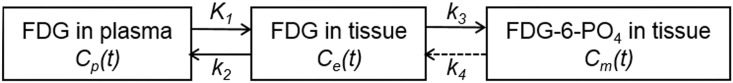
Three-compartment model of FDG.

TACs were derived from ROIs in the series of reconstructed images. The TAC of the blood-pool and the Tissue TAC were utilized as the input and output functions to fit a standard three-compartment model [20] (Fig 1) using the Levenberg-Marquardt algorithm to assess FDG kinetics in tissue [27].

Here C_p_(t) is the plasma activity, and C_e_(t) and C_m_(t) are the concentrations of non-metabolized (free) and phosphorylated (bound) FDG inside the tissue. *K*_*1*_, *k*_*2*_, *k*_*3*_, *k*_*4*_ are rate constants. *K*_*1*_ (mL per second per gram) is the forward rate constant from blood to tissue compartment. *k*_*2*_ (per second) is the reverse rate constant between those two compartments, *k*_*3*_ (per second) shows the phosphorylation rate of FDG to FDG-6-PO_4_ by hexokinase, and *k*_*4*_ (per second) represents the dephosphorylation of FDG-6-PO_4_.

The kinetic of the tracer depicted in Fig 1 is mathematically expressed as in the following equations:

$$\frac{dC_{e}(t)}{dt}\;=\;K_{1}\;\cdot \;C_{p}(t)\;-\;(k_{2}\;+\;k_{3})\;\cdot \;C_{e}(t)\;+\;k_{4\;}\cdot \;C_{m}(t),$$ (3)

$$\frac{dC_{m}(t)}{dt}\;=\;K_{3}\;\cdot \;C_{e}(t)\;-\;k_{4\;}\cdot \;C_{m}(t),$$ (4)

$$C_{T}(t)\;=\;[\;C_{e}(t)\;+\;C_{m}(t)\;]\;+\;V_{B\;}\cdot \;C_{p}(t),$$ (5)

*V*_*B*_ is a coefficient representing vascular volume. C_T_(t) represents the time-activity data of the tissue observed from PET images, which can be solved to the following form [28]

$$C_{T}(t)\;=\;\frac{k_{1}}{\alpha_{2}\;-\;\alpha_{1}}\;[\;(k_{3}\;+\;k_{4}\;-\;\alpha_{1})\;e^{-\;\alpha_{1}t}\;+\;(\alpha_{2}\;-\;k_{3}\;-\;k_{4})\;e^{-\;\alpha_{2}t}\;]⊗\;\;C_{p}(t)\;+\;V_{B}\;\cdot \;C_{p}(t)$$ (6)

(⊗ stands for convolution), where

$$\alpha_{1,2}\;=\;\frac{k_{2}\;+\;k_{3}\;+\;k_{4}\;\mp \;\sqrt{{\left(k_{2}\;+\;k_{3}\;+\;k_{4}\right)}^{2}\;-\;4k_{2}k_{4}}}{2}$$ (7)

Therefore, with the input function C_p_(t) and the output function C_T_(t), parameters *K*_*1*_, *k*_*2*_, *k*_*3*_, *k*_*4*_ can be obtained by fitting the experimental data to Eq 6.

The influx rate constant *K*_*i*_ was determined by

$$K_{i}=\;\frac{K_{1}k_{2}}{k_{2\;}+\;k_{3}}$$ (8)

### Histologic Examination of Inflamed Tissue and Tumors

After PET data acquisition, animals were sacrificed. For different groups, different tissues (tumor, lung, liver and gastrocnemius muscle) were carefully excised from the body. The lung was soaked in 50% Optimum Cutting Temperature Compound (OCT) for 30 min in advance. The tissues were mold in OCT cryofixative and frozen in liquid nitrogen for 15 seconds, and then transferred to the -80°C refrigerator for 12 hours. Tissue sections 10 um thick were cut and collected on glass slides. The sections were fixed in 4% paraformaldehyde and stained with hematoxylin and eosin (H&E staining) to visualize tissue morphology.

### Statistical Analysis

With both the semi-quantitative SUV and quantitative *K*_*i*_, we performed statistical analysis. All results were expressed as mean ± SD. The mean of all interested parameters such as SUVmax and *K*_*i*_ in each study was compared via Kruskal-Wallis non-parametric (KW) test among all the 6 groups. Once the Kruskal-Wallis non-parameteric test shows significant difference between the groups in the study, a post-hoc way multiple pairwise comparisons was conducted on every two subgroups to determine whether they are statistically different [29]. A *p*-value less than 0.05 were considered statistically significant.

## Results

### I. Histological Results of Animal Models

**Fig 2:**
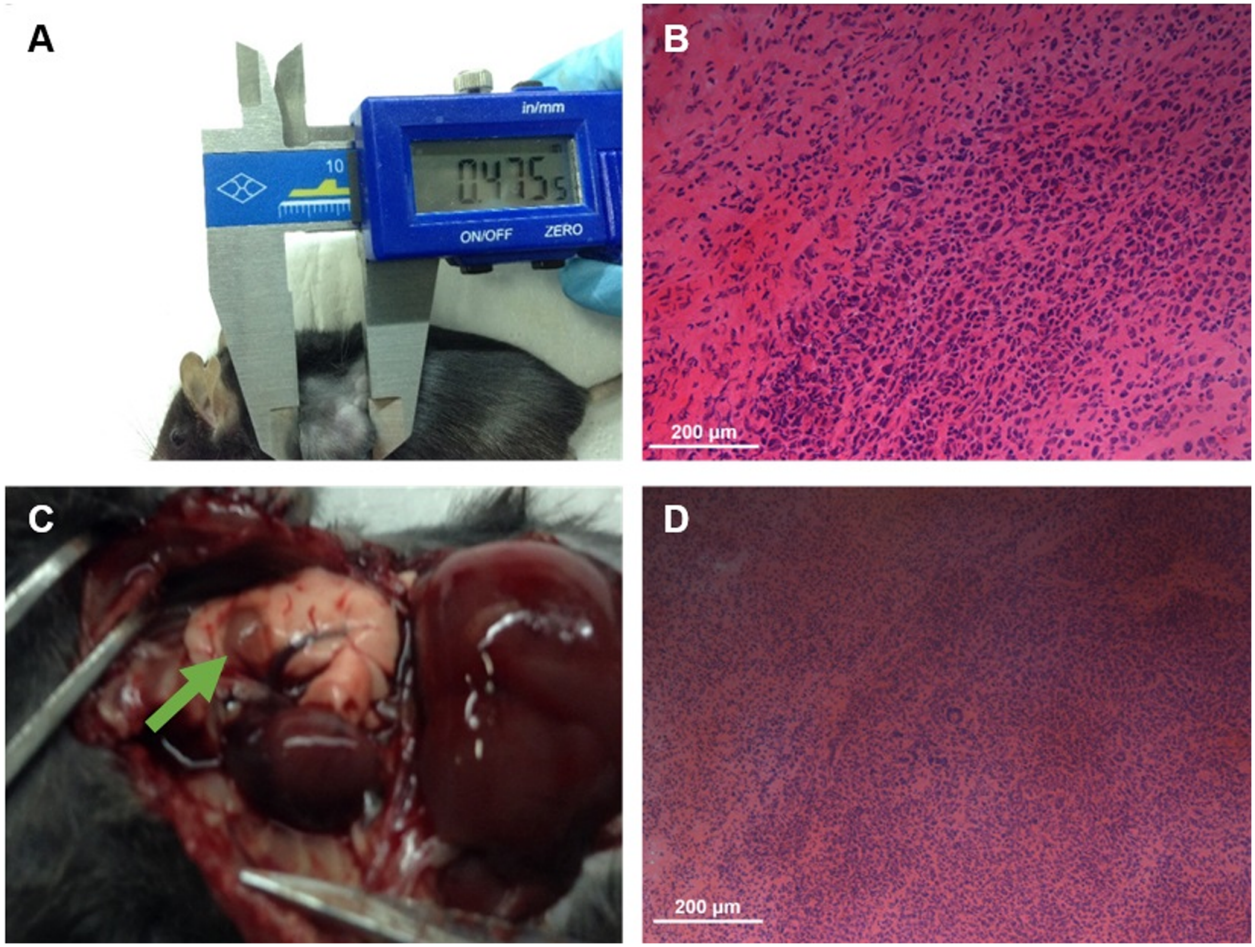
Anatomic structure and H&E stained sections of tumor lesions. (A), (B) Subcutaneous tumor. (C), (D) In situ tumor.

**Fig 3:**
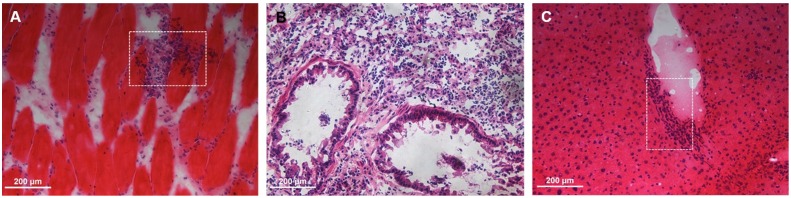
H&E stained sections of inflammatory lesions. (A) Subcutaneous inflammation. (B) In situ inflammation. (C) Spontaneous inflammation.

The image and tissue morphology of tumors are illustrated in Fig 2. From the images (Fig 2A and 2C), the size and shape of both subcutaneous and in situ tumor are evident. The tissue morphology shows the pleomorphic and hyperchromatic nuclei as well as regions of increased cell density (Fig 2B and 2D). For inflammatory lesions in different locations, as seen in Fig 3, massive inflammatory infiltration of neutrophils is seen in and between muscle fibers (Fig 3A), pulmonary tissue (Fig 3B) and liver tissue (Fig 3C).

The histological results indicate that the animal models of both tumor and inflammation are successfully built and can be regarded a verification of PET imaging data.

### II. Visual Analysis

**Fig 4:**
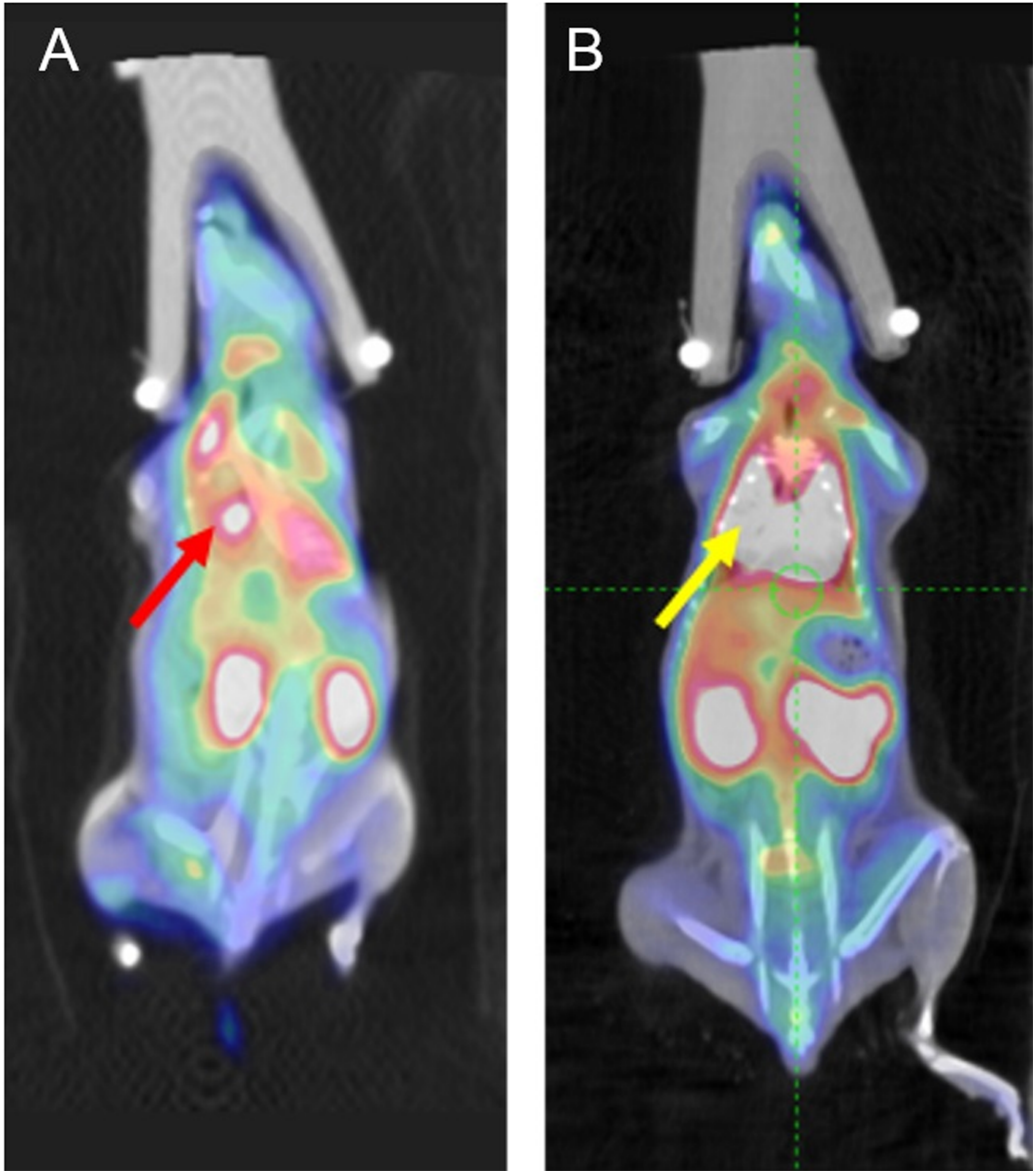
Examples of visual analysis. (A) In situ tumor. Red arrow: high FDG uptake caused by tumor inside the lung (value of SUV was around 1.7) (B) In situ inflammation. Yellow arrow: high FDG uptake caused by inflammation inside the lung (value of SUV was also around 1.7)

Examples of visual analysis (in situ tumor and inflammation) are illustrated in Fig 4. From both in situ tumor and inflammation groups, a high FDG accumulation can be observed inside the lung (Fig 4A, red arrow and Fig 4B, yellow arrow) with the corresponding SUV value as 1.7. Therefore from the static data, it is hard to differentiate inflammatory lesions from malignancies via visual analysis from the PET image alone.

### III. Analysis of data from different lesions at the same location of the body

Since no clear differences were observed from visual analysis alone, results from static analysis (SUVmax) and kinetic analysis (*Ki*) of tumor and inflammation at the same location were compared (subcutaneous and in situ). For subcutaneous lesions, results from both static and kinetic analysis showed significant differences. For in situ lesions, no obvious difference was found between SUVmax values of tumor and inflammation while *Ki* values showed statistical differences.

#### Static Analysis

**Fig 5:**
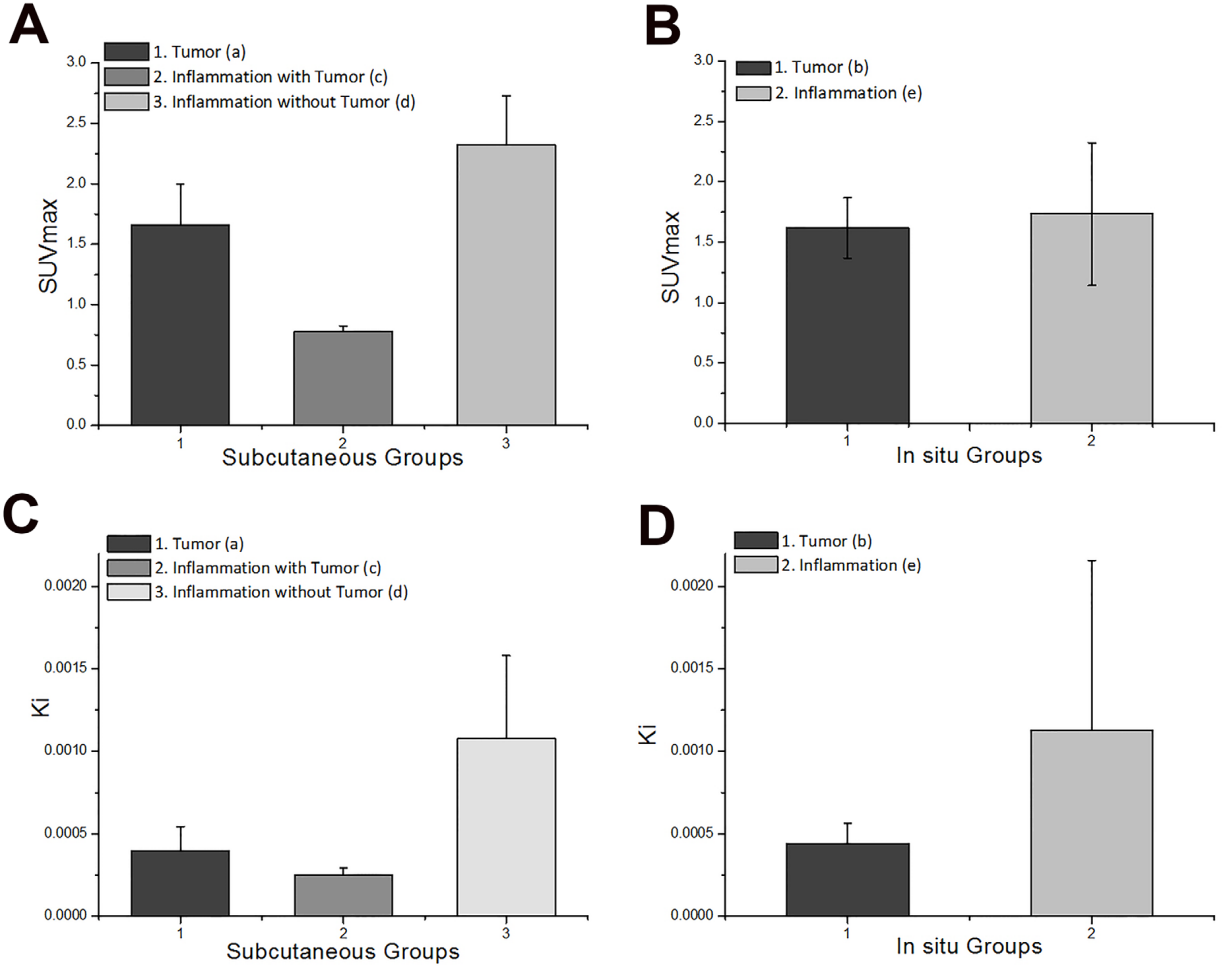
Comparison of SUVmax and *Ki* in tumors and inflammations from the same body location. (A) SUVmax from subcutaneous groups. (B) SUVmax from in situ groups. (C) *Ki* from subcutaneous groups. (D) *Ki* from in situ groups.

Fig 5A shows the comparison of SUVmax from 3 subcutaneous groups: tumor, inflammation **with** tumor and inflammation **without** tumor. Inflammation without tumor group had the highest SUVmax (2.32 ± 1.00), followed by tumor (1.66 ± 0.34) and inflammation with tumor (0.78 ± 0.05, Table 2). A significant difference in SUVmax between three groups was observed (*p*<0.01). Among the subcutaneous groups, the FDG uptake of inflammation is much higher without the existence of tumor than that of inflammation when both tumor and inflammatory lesions take place.

**Table 2:** Values of SUVmax from each group.

Group	SUVmax
Subcutaneous tumor	1.66 ± 0.34
In situ tumor	1.62 ± 0.25
Subcutaneous inflammation **with** tumor	0.78 ± 0.05
Subcutaneous inflammation **without** tumor	2.32 ± 0.43
In situ inflammation	1.73 ± 0.59
Spontaneous liver inflammation	1.40 ± 0.32

When it comes to in situ lesions (Fig 5B), mean values SUVmax of inflammation (1.73 ± 0.59) is slightly higher than that of tumor (1.62 ± 0.25, Table 2). No significant difference between two groups was found through KW test (*p* = 1). The current results from static analysis failed to distinguish in situ tumor and inflammation.

#### Kinetic Analysis

The result of kinetic analysis for subcutaneous groups is shown in Fig 5C. Similar to static analysis, values of *Ki* were highest among inflammation without tumor (mean value 0.001 mL/s/g), followed by tumor (mean value 3.95×10^−4^ mL/s/g) and inflammation with tumor (mean value 2.49×10^−4^ mL/s/g, Table 3). Statistical analysis also showed significant difference between these three groups (*p*<0.005). Consistency between static and kinetic analysis for subcutaneous groups indicates that both static and kinetic analysis can be used to differentiate subcutaneous tumor and inflammation.

**Table 3:** Values of influx rate constant *Ki* from kinetic analysis.

Group	K_1_ (mL/s/g)	k_2_ (s^-1^)	k_3_ (s^-1^)	k_4_ (s^-1^)	*Ki* ^a^ (mL/s/g)
Subcutaneous tumor	0.00229	0.042	0.0104	0.00177	0.000395
In situ tumor	0.185	0.459	0.00166	0.00499	0.000438
Subcutaneous inflammation **with** tumor	0.00326	0.0124	0.00119	0.000706	0.000249
Subcutaneous inflammation **without** tumor	0.00531	0.0578	0.19	0.00993	0.00108
In situ inflammation	0.0241	0.674	0.148	0.56	0.00112
Spontaneous liver inflammation	0.00817	0.00982	0.109	0.418	0.0033

^a^Influx rate constant *Ki* were calculated from estimated parameter values as (K_1_k_3_) / (k_2_ + k_3_)

The result of kinetic analysis from in situ groups was in sharp contrast to the finding that static analysis cannot distinguish in situ lesions. As illustrated in Fig 5D, values of *Ki* from inflammation group (mean value 0.00112 mL/s/g) were much higher than values from tumor group (mean value 4.38×10^−4^ mL/s/g, Table 3) (KW test *p*<0.001).

### IV. Analysis of data of the same type of lesion from different locations of the body

In addition to different lesions at the same body location, SUVmax and *Ki* values of tumors or inflammations from different body locations were compared. Results from static and kinetic analysis are generally similar. For tumors, values from different locations showed no significant differences. For inflammatory lesions, differences were found in all four groups according to KW test.

#### Static Analysis

**Fig 6:**
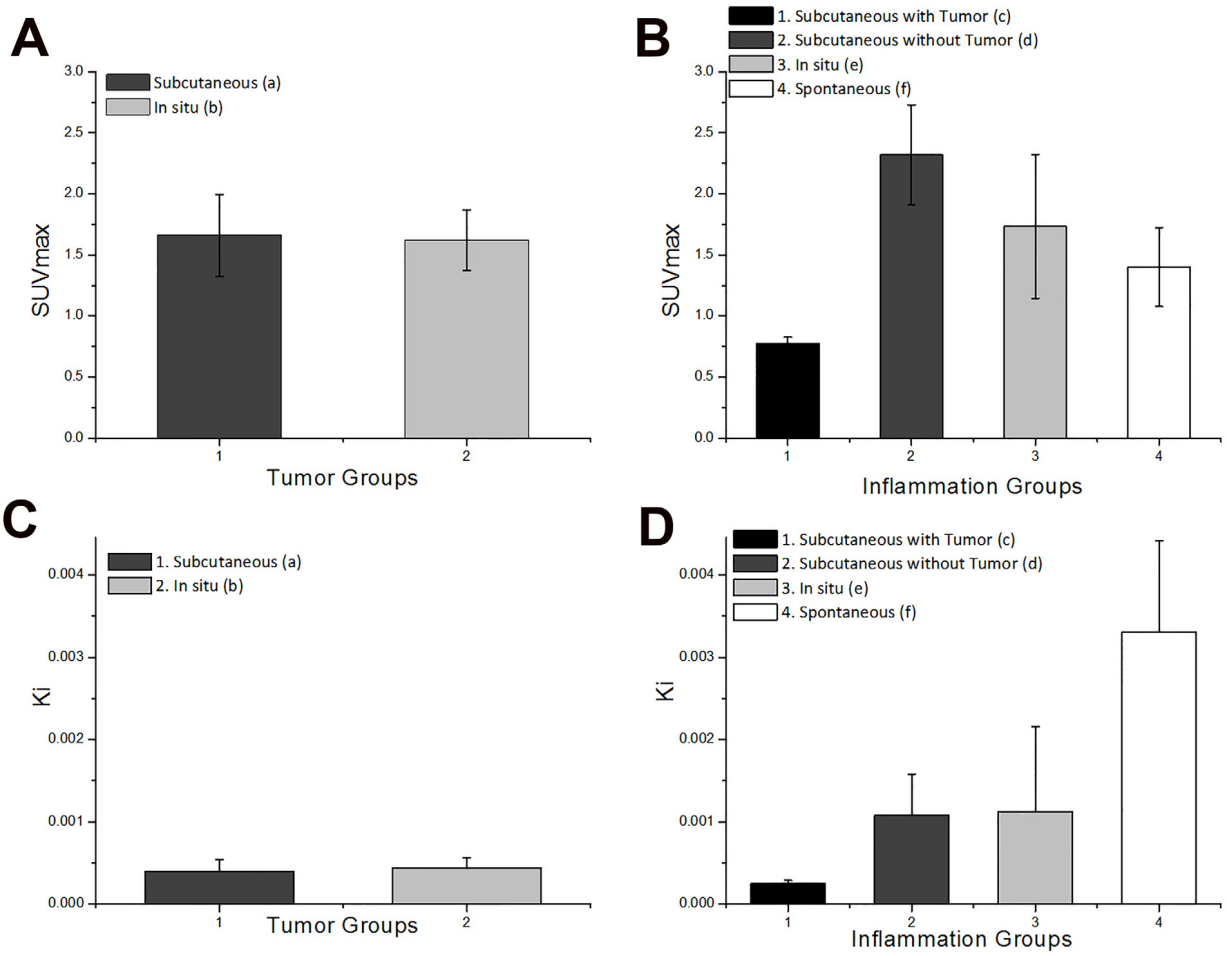
Comparison of SUVmax and *Ki* in tumor or inflammation from different body locations. (A) SUVmax from tumor groups. (B) SUVmax from inflammation groups. (C) *Ki* from tumor groups. (D) *Ki* from inflammation groups.

**Fig 7:**
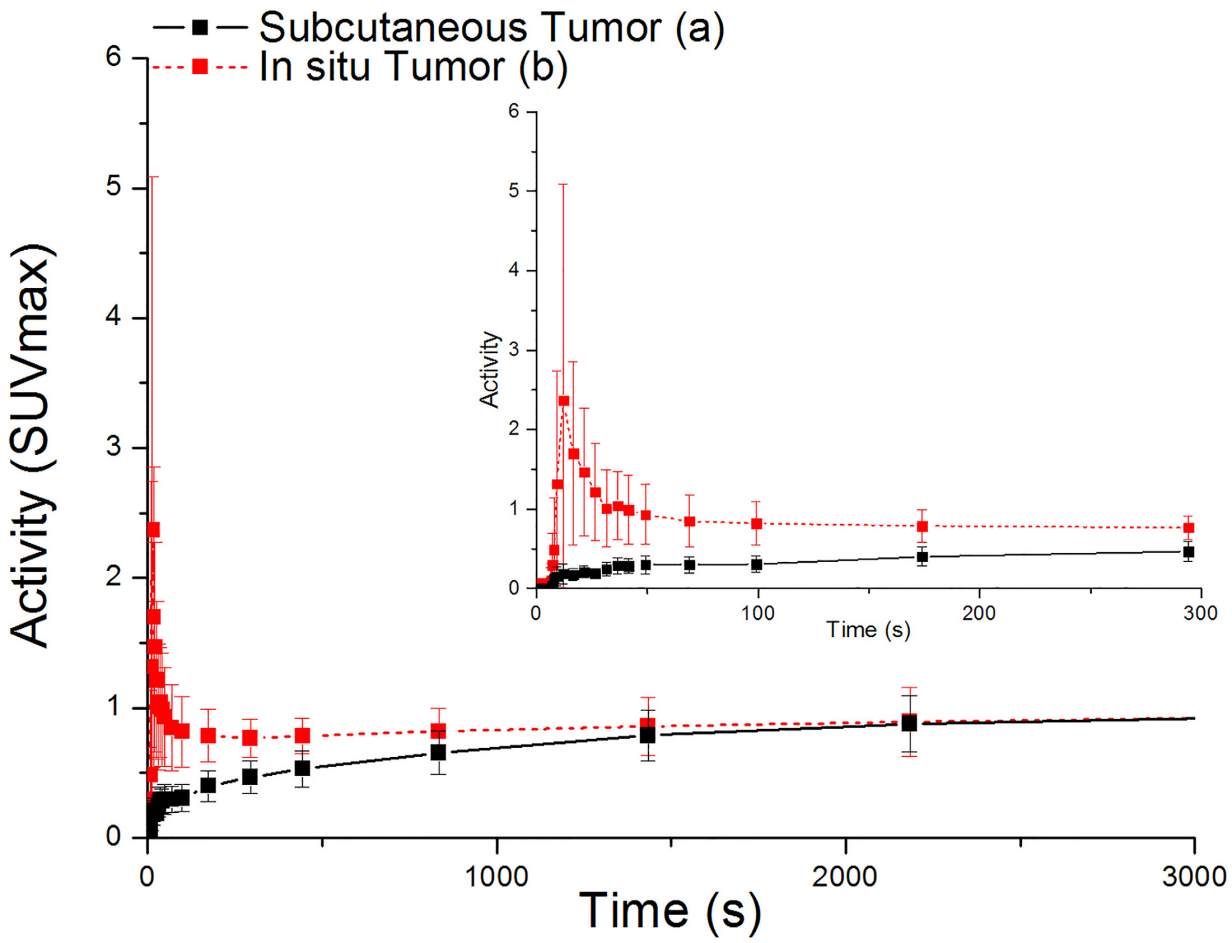
Time Activity Curves of subcutaneous tumor and in situ tumor. (From 0 to 3000s and an expanded curve from 0 to 300 s)

For tumors from different locations of the body (Fig 6A), values of SUVmax showed no obvious difference (*p* = 0.91) between subcutaneous groups (1.66 ± 0.34) and in situ groups (1.62 ± 0.25, Table 2). However, differences of these two groups can be observed from TAC visually (Fig 7).

As for inflammation located in different parts of the body, it is shown in Fig 6B that SUVmax values of inflammation were highest among subcutaneous groups without tumor (2.32 ± 1.00), followed by in situ inflammation (1.73 ± 0.59), spontaneous inflammation (1.40 ± 0.32) and subcutaneous inflammation with tumor (0.78 ± 0.05, Table 2). Significant statistical difference were found in these four groups through KW test (*p*<0.005). Each two-group pair was compared by the multiple pairwise comparisons after KW test [29]. The subcutaneous inflammation with tumor group showed significant difference with the situ inflammation (*p* = 0.03) and subcutaneous inflammation without tumor (*p* = 0.002). However, there was no significant difference between inflammation without tumor, in situ inflammation and spontaneous inflammation.

#### Kinetic Analysis

In accord with static data, there were no significant difference of *Ki* values (*p* = 0.46, Fig 6C) between subcutaneous (mean value 3.95×10^−4^ mL/s/g) and in situ tumor groups (mean value 4.38×10^−4^ mL/s/g, Table 3). Therefore, from our current results, tumors from different body locations cannot be differentiated. However, the shapes of TACs from subcutaneous and in situ groups are quite different from each other (Fig 7).

Fig 6D shows the result of kinetic analysis from inflammation among different body locations. Spontaneous inflammation has the highest *Ki* values (mean value 0.033 mL/s/g), followed by in situ inflammation (mean value 0.00112) and subcutaneous inflammation without tumor (mean value 0.00108) respectively. Same as static results, subcutaneous inflammation with tumor has lowest mean *Ki* value of 2.49×10^−4^ mL/s/g (Table 3). For statistical analysis, all four groups showed significant difference as a whole according to KW test (*p* = 0.01). Statistical results from the multiple pairwise comparisons after KW test presented that the subcutaneous inflammation with tumor has the lowest Ki values than the spontaneous inflammation with *p* = 0.011 [29]. Other three groups showed no significant differences between each other.

## Discussion

In this study, we examined the hypothesized higher sensitivity of the tracer kinetic modeling approach in differentiating in situ inflammation and tumor. Indeed, we found that, while SUVmax failed to detect any difference between in situ inflammation and tumor, the *Ki* values succeeded in doing so.

It is reported that, in inflammatory lesions in lungs, *Ki* is closely related to neutrophil activation with pulmonary sequestration or infiltration and that the correlation of SUV and *Ki* was low [30]. Consistently, we also found weak correlation of SUV and *Ki* when comparing in situ tumor with inflammation (R^2^ = 0.05, *p* = 0.718 and R^2^ = 0.007, *p* = 0.831, respectively), which was in contrast to the relatively higher correlation between these two measurements when assessing subcutaneous groups or inflammatory lesions in different locations of the body. Thus, quantifying absolute FDG uptake, instead of the semi-quantification, in lungs is more adequate and sensitive.

In our study, inflammatory lesions from different parts of the body presented high values of SUVmax and *Ki*. The reason of high FDG uptake for inflammatory lesions is that inflammatory cells such as macrophages and neutrophils had the elevated expression of glucose transporters (GLUTs) such as GLUT-1 and GLUT-3 [31]. Besides that, Cytokines and growth factors played important roles in promoting the affinity of glucose transporters for deoxyglucose. This kind of phenomenon has not been seen in tumors [17, 32]. We found that inflammation in different situation has different kind of FDG uptake. Assessing the kinetics of inflammations from different part of the body might help establishing a new way to evaluate inflammation.

Based on the result of the data, the uptake of inflammation appeared to be the highest without the existence of tumor. However, co-existence of inflammation and tumor caused a significant reduction of the uptake of inflammatory lesions, which was far lower than tumor uptake alone. Mochizuki et al. found that both tumor and inflammatory lesions expressed more GLUT-1 and GLUT-3, which is the main cause of high FDG uptake in tumor and inflammatory lesions. Their study also discovered that the level of GLUT-1 expression in tumor was significantly higher than that in inflammation [33]. Mamede et al. studied how inflammation affects the FDG uptake in tumor tissues as a whole. They found that the contribution of inflammation to the overall FDG uptake in NSCLC is not significant, and hypothesized that inflammatory cells take FDG not using only the expression of GLUT-1, but using the expression of other glucose transporters or hexokinases [34]. The mechanisms of FDG accumulation in inflammations still remain unclear. Based on their results, we supposed the high expression of GLUT-1 in tumor cells may reduce GLUT-1 expression of inflammatory cells, when tumor and inflammation co-exist. Therefore, the FDG uptake will be reduced. Apparently our current study was not designed to understand the mechanism of this observation and additional study is needed for such examination.

When it comes to tumor in different parts of the body, usually tumors were planted subcutaneously in mice for convenience, low cost and reproducibility. However, recent studies have shown that orthotopic location of tumor may be more capable of imitating the real-life situation [35]. In our current studies, although we were not able to differentiate tumors in different locations according to values of SUVmax or *Ki*, subcutaneous and in situ tumors showed huge difference in time activity curve. The FDG uptake of in situ tumors showed high initial activity with a downward trend, while subcutaneous tumor showed gradual accumulation of FDG. The activities of both subcutaneous and in situ tumor end up at almost the same level. Graves et al. reported that although both in situ and subcutaneous tumor may share elevated metabolism and glycolytic activity based on the result of FDG-PET, subcutaneous tumors showed significant hypoxia while in situ tumors were well-oxygenated and the dynamic range remained unknown [36]. The difference of hypoxia might be one of cause for differences in time activity from tumors in different locations. For different inflammations, our results showed significant difference between inflammations in different body locations, which indicates that in situ inflammation models cannot be replaced by subcutaneous ones regardless of its easy procedures.

In FDG compartment modeling, when the dephosphorilization is so slow that the tracer uptake is irreversible, the rate constant k_4_ can be assumed to be zero [20]. For FDG undergoing irreversible trapping, the analyzing method can also be simplified using the Patlak graphical analysis [37]. However, we observed significant non-zero k_4_ in our work during experimental time for all animals (*p* = 0.0018). In inflammatory cell, the levels of glucose-6-phosphatase are higher than that in malignant cells in a previous study which discussed the malignant and inflammatory process differences [15]. In our work, the dephosphorilation rate (k_4_) in situ inflammation group is almost significantly higher than that in tumor group (*p* = 0.0532) with even the small number of samples used in our study. In the subcutaneous groups (subcutaneous inflammation with tumor, subcutaneous inflammation without tumor and subcutaneous tumor), the dephosphorilation rate (k_4_) is significantly different (*p* = 0.0395). For each subgroup, k_4_ in subcutaneous inflammation without tumor is significant higher than that of subcutaneous inflammation with tumor (*p* = 0.0209). Based on our data analysis, the dephosphorilation rate (k_4_) is non-ignorable to simplify the model most likely due to the inclusion of the inflammation animals.

There were limitations in our current study. The major one is the small sample size. We attempted to address this issue by using the non-parametric tests. Future studies with larger sample sizes are needed to confirm our findings. Secondly, our data acquisition was long for small animals and also not practical for clinical settings. We will evaluate the use of shorter scan for adequately distinguishing inflammation from tumors. Thirdly, the mouse model we used (LLC) is one type of NSCLC model. Therefore, the results might not be applicable in the situation of Small Cell Lung Carcinoma (SCLC).

## Conclusions

Dynamic FDG-PET imaging is more sensitive to quantify FDG uptake in orthotopic lesions and to differentiate in situ malignancies from inflammations than static analysis for NSCLC. And comparison of influx rate constant *Ki* can be a metric to assess inflammations at different part of the body.

## Supporting Information

S1 File Supplementary Data of Fig 5, Fig 6 and Fig 7. Supplementary Data of Fig 5 **(Table A)**; Supplementary Data of Fig 6 **(Table B)**; Supplementary Data of Fig 7 **(Table C)**.
(PDF)
