# Supplementary material for: Dynamic FDG-PET Imaging to Differentiate Malignancies from Inflammation in Subcutaneous and In Situ Mouse Model for Non-Small Cell Lung Carcinoma (NSCLC)
Source: PLoS One. 2015 Sep 30;10(9):e0139089. doi: 10.1371/journal.pone.0139089 (PMC4589399; doi:10.1371/journal.pone.0139089)
Supplement: S1 File — Supplementary Data of Fig 5 (Table A); Supplementary Data of Fig 6 (Table B); Supplementary Data of Fig 7 (Table C). (PDF) [file pone.0139089.s001.pdf]

Supporting Information

Table A. Supplementary Data of Fig. 5

SUVmax from Subcutaneous Groups											
						Mean	Standard Deviation				
Tumor(a)	1.9	1.9	1.4	1.9	1.2	1.66	0.33615				
Inflammation with Tumor(c)	0.8	0.7	0.8	0.8	--	0.775	0.05				
Inflammation without Tumor(d)	2.5	2.8	1.8	2.2	--	2.325	0.427200187265875				
Ki from Subcutaneous Groups											
						Mean	Standard Deviation				
Tumor(a)	3.59E-04	6.44E-04	3.78E-04	2.46E-04	3.47E-04	3.95E-04	1.48E-04				
Inflammation with Tumor(c)	2.74E-04	2.14E-04	2.93E-04	2.14E-04	--	2.49E-04	4.10E-05				
Inflammation without Tumor(d)	0.0018	9.90E-04	8.81E-04	6.35E-04	--	0.00108	5.04E-04				
SUVmax from in situ Groups											
										Mean	Standard Deviation
Tumor(b)	1.6	2	1.3	1.6	1.6	--	--	--	--	1.62	0.249
Inflammation (e)	1.5	1.3	1.7	1.1	1.8	1.6	1.3	3	2.3	1.73333	0.58949
Ki from in situ Groups											
										Mean	Standard Deviation
Tumor(b)	5.94E-04	3.98E-04	2.98E-04	5.39E-04	3.65E-04	--	--	--	--	4.39E-04	1.24E-04
Inflammation (e)	0.00119	7.76E-04	6.03E-04	9.49E-04	1.32E-04	9.66E-04	9.18E-04	8.23E-04	0.00377	0.00112	0.00103

Table B. Supplementary Data of Fig. 6

SUVmax from Tumor Groups											
						Mean	Standard Deviation				
Subcutaneous (a)	1.9	1.9	1.4	1.9	1.2	1.66	0.33615				
in situ (b)	1.6	2	1.3	1.6	1.6	1.62	0.249				
Ki from Tumor Groups											
						Mean	Standard Deviation				
Subcutaneous (a)	3.59E-04	6.44E-04	3.78E-04	2.46E-04	3.47E-04	3.95E-04	1.48E-04				
in situ (b)	5.94E-04	3.98E-04	2.98E-04	5.39E-04	3.65E-04	4.39E-04	1.24E-04				
SUVmax from Inflammation Groups											
										Mean	Standard Deviation
Subcutaneous with Tumor (c)	0.8	0.7	0.8	0.8	--	--	--	--	--	0.775	0.05
Subcutaneous without Tumor (d)	2.5	2.8	1.8	2.2	--	--	--	--	--	2.325	0.427200187265876
in situ (e)	1.5	1.3	1.7	1.1	1.8	1.6	1.3	3	2.3	1.73333	0.58949
Spontaneous (f)	1.1	1.6	1.1	1.5	1.2	1.9	--	--	--	1.4	0.32249
Ki from Inflammation Groups											
										Mean	Standard Deviation
Subcutaneous with Tumor (c)	2.74E-04	2.14E-04	2.93E-04	2.14E-04	--	--	--	--	--	2.49E-04	4.10E-05
Subcutaneous without Tumor (d)	0.0018	9.90E-04	8.81E-04	6.35E-04	--	--	--	--	--	0.00108	5.04E-04
in situ (e)	0.00119	7.76E-04	6.03E-04	9.49E-04	1.32E-04	9.66E-04	9.18E-04	8.23E-04	0.00377	0.00112	0.00103
Spontaneous (f)	0.00176	0.00343	0.00363	0.00397	0.00224	0.00477	--	--	--	0.0033	0.00112

Table C. Supplementary Data of Fig. 7

Time Activity Curves of subcutaneous tumor and in situ tumor (SUVmax)					
in situ Tumor			Subcutaneous Tumor		
Time (s)	SUVmax (mean)	Standard Deviation	Time (s)	SUVmax (mean)	Standard Deviation
1.5	0.06951	0	1.5	8.43E-16	0
3.25	2.03E-11	4.54E-11	3.25	0	0
3.75	1.40E-16	1.93E-16	3.75	0	0
4.25	1.56E-10	3.50E-10	4.25	4.29E-15	0
4.75	7.08E-04	0.00158	4.75	4.29E-15	0
5.25	2.52E-07	5.63E-07	5.25	0.00102	0.00145
5.75	0.02623	0.05865	5.75	0.00102	0.00145
6.25	0.07949	0.17773	6.25	0.00557	0.00764
6.75	0.10077	0.21796	6.75	0.00564	0.00667
7.25	0.29294	0.40338	7.25	0.0345	0.03443
7.75	0.48641	0.6517	7.75	0.12112	0.06612
8.25	1.31419	1.42823	8.25	0.15093	0.11669
12.25	2.36777	2.7188	12.25	0.18381	0.12404
17.25	1.70001	1.1548	17.25	0.17664	0.07712
22.25	1.46774	0.80694	22.25	0.20934	0.07471
27.25	1.21343	0.60589	27.25	0.19098	0.05832
32.25	1.00811	0.48625	32.25	0.24261	0.08461
37.25	1.04372	0.42419	37.25	0.28633	0.10628
42.25	0.98922	0.43585	42.25	0.28271	0.09281
49	0.9308	0.37872	49	0.29878	0.11502
69	0.8481	0.32997	69	0.2986	0.1028
99	0.82025	0.27188	99	0.30748	0.10378
174	0.78752	0.20153	174	0.40045	0.11879
294	0.76596	0.14685	294	0.46717	0.12634
444	0.78534	0.13392	444	0.5335	0.13916
834	0.8227	0.17685	834	0.6563	0.16715
1434	0.85939	0.22565	1434	0.78796	0.19735
2184	0.89444	0.2659	2184	0.87915	0.21801
3084	0.92486	0.29821	3084	0.92194	0.2245
